# Molecular characterization of *bla*_ESBL_–harboring conjugative plasmids identified in multi-drug resistant *Escherichia coli* isolated from food-producing animals and healthy humans

**DOI:** 10.3389/fmicb.2013.00188

**Published:** 2013-07-11

**Authors:** Juan Wang, Roger Stephan, Maria Karczmarczyk, Qiongqiong Yan, Herbert Hächler, Séamus Fanning

**Affiliations:** ^1^UCD Centre for Food Safety, School of Public Health, Physiotherapy and Population Science, UCD Centre for Molecular Innovation and Drug Discovery, University College DublinDublin, Ireland; ^2^Institute for Food Safety and Hygiene, Vetsuisse Faculty, University of ZurichZurich, Switzerland

**Keywords:** ESBLs, *E. coli*, horizontal gene transfer, replicon typing, conjugation, S1-nuclease PFGE, plasmid profiling, plasmids

## Abstract

**Background:** Extended-spectrum β-lactamase (ESBL)-encoding genes are frequently mapped to plasmids, yet few of these structures have been characterized at the molecular level, to date.

**Methods:** Eighty-seven ESBL-producing *Escherichia coli* were isolated from fecal samples of food-producing animals and healthy humans in Switzerland from 2009 to 2011. Plasmid DNA of all isolates was purified. Broth mating assays were carried out individually for 32 isolates to determine if the ESBL marker could be transferred by conjugation. The plasmid sizes were determined by S1-nuclease pulsed-field gel electrophoresis (PFGE) and the plasmids were typed by PCR-based replicon typing. Susceptibility tests by disk diffusion followed with a re-analysis S1-nuclease PFGE and PCRs were performed to confirm plasmid transfer. Microarray was performed to detect additional antibiotic resistance markers and multi-locus sequence typing was also performed in selected donor strains. The phylotypes were identified by triplex PCR.

**Results:** About half (*n* = 46) of the 87 isolates carried small (<20-kb) plasmids. All selected 32 isolates contained large plasmids (ranging in sizes from 20- to 600-kb). Eleven plasmid replicon types were detected. Of these, IncFIA (*n* = 5), IncFIB (*n* = 9), and IncK/B (*n* = 4) were common. Nine isolates demonstrated the ability to transfer their cefotaxime resistance marker at high transfer rates. Plasmid profile re-analysis of these transconjugants identified 16 plasmids. IncFIB and IncI1 were the most prevalent replicon types. Phylogenetic grouping showed that five of the nine donor strains belonged to phylogroup B1. Nine different sequence types were identified in nine tested donor strains.

**Conclusion:** Characterization of these ESBL-encoding conjugative plasmids extends our understanding on these resistance markers in multi-drug resistant *E. coli* cultured from healthy human and animal sources.

## INTRODUCTION

Global dissemination of multiple antibiotic resistance and virulence traits by plasmids poses an increasing threat to the successful treatment of bacterial infectious diseases in animals and humans alike (Bush, 2010). Extended-spectrum β-lactamase (ESBL) producing *Escherichia coli* has become one of the most important causes of nosocomial and community acquired infections (Coque et al., 2008; Foxman, 2013) and most of the ESBL enzymes recognized are expressed from genes mapped to plasmids (Paterson and Bonomo, 2005; Garcillan-Barcia et al., 2011). It is acknowledged that the horizontal transfer of plasmids carrying these ESBL genes is an important contributory factor in the epidemiology of this bacterial ecosystem (EFSA Panel on Biological Hazards [BIOHAZ], 2011). The predominant ESBL families of clinical importance include TEM, SHV, and CTX-M (Bush and Jacoby, 2010); and the genes conferring this resistance phenotype in animals include *bla*_CTX-M-1_ (the most frequently identified ESBL), *bla*_CTX-M-14_, *bla*_TEM-52_, and *bla*_SHV-12_ (EFSA Panel on Biological Hazards [BIOHAZ], 2011). *E. coli* is the most common bacterial species identified with these genes.

Plasmids evolve as an integral part of the bacterial genome, consisting of several extra-chromosomal traits, one of which is their resistance genes, which can be exchanged among bacteria of different origins by conjugation (Carattoli, 2011). Those expressing an ESBL phenotype frequently carry genes encoding resistance to other commonly used antimicrobial drug classes (such as aminoglycosides, chloramphenicols, fluoroquinolones, or tetracycline; Harajly et al., 2010; Rogers et al., 2011). Plasmid-mediated transfer of drug-resistance genes among various bacterial species is considered to be one of the most important mechanisms driving the spread of multi-drug resistance (MDR) markers. The use of antimicrobial compounds in human and veterinary medicine also constitutes a risk factor for the selection and dissemination of resistant clones, and also plasmids containing the corresponding resistance genes. Consequently, these features limit the treatment options available when ESBL-producing organisms are encountered. Therefore, as a first step to address this problem, it is important to monitor the dissemination of ESBL-producing bacteria, characterizing these microbes when they arise. Moreover, studies describing the genetic basis of plasmids derived from bacteria recovered from animals, humans, and the environment are essential.

Extended-spectrum-β-lactamase transmission is mainly driven by insertion sequences, transposons, integrons, and plasmids, some of which are homologous in isolates from both food producing animals and humans (van Hoek et al., 2011; Stalder et al., 2012). Plasmid-encoded genes can arise from multiple sources (Boyd et al., 1996) being subsequently disseminated by horizontal gene transfer (HGT). HGT is responsible for the dissemination of many traits associated with bacteria, including antibiotic resistance and virulence. In addition, broad-host-range plasmids play an important role in bacterial adaptation to new environments. Taken together these characteristics provide much of the motivation of extending our understanding of the structural relationships that exist between plasmids from a variety of bacterial sources. Following the dissemination routes of antibiotic resistance genes on ESBL-harboring plasmids is important to broaden our knowledge of how these genes are shared horizontally across species boundaries as well as their evolution (Zhou et al., 2012). Moreover, characterization based on plasmid profiling and the definition of corresponding incompatibility (Inc) group is an integral part of plasmid epidemiological surveillance enhancing discrimination between *E. coli* strains (Carattoli et al., 2005).

In this paper, we report on the preliminary characterization of ESBL-producing *E. coli* isolates that were originally described following culture and phenotypic studies in healthy humans and animals in Switzerland. The aims of this study were (i) to identify the plasmids relating to ESBL markers, (ii) to characterize the donor *E. coli* isolates by phylogenetic grouping and multi-locus sequence typing (MLST), and (iii) to determine the replicon types and S1 nuclease-based plasmid profiles, following pulsed-field gel electrophoresis (PFGE). The epidemiological data presented extends our understanding of this resistance type in humans and animals.

## MATERIALS AND METHODS

### BACTERIAL STUDY STRAINS COLLECTIONS

Extended-spectrum-β-lactamase-producing *E. coli* (*n* = 87) were cultured from fecal samples of healthy humans (*n* = 34) and healthy food-producing animals (*n* = 52) in Switzerland, as well as one from a mastitis milk sample. To prevent sample clustering, at most two animal samples per farm were taken throughout Switzerland. All human samples were collected in urban areas, and each individual was tested only once. Prior to testing, all isolates were streaked for purity on MacConkey agar (Oxoid, Basingstoke, England). The protocols for collecting and isolating the strains were described previously (Geser et al., 2012a, b).

### PLASMID PROFILING

**Figure A1:**
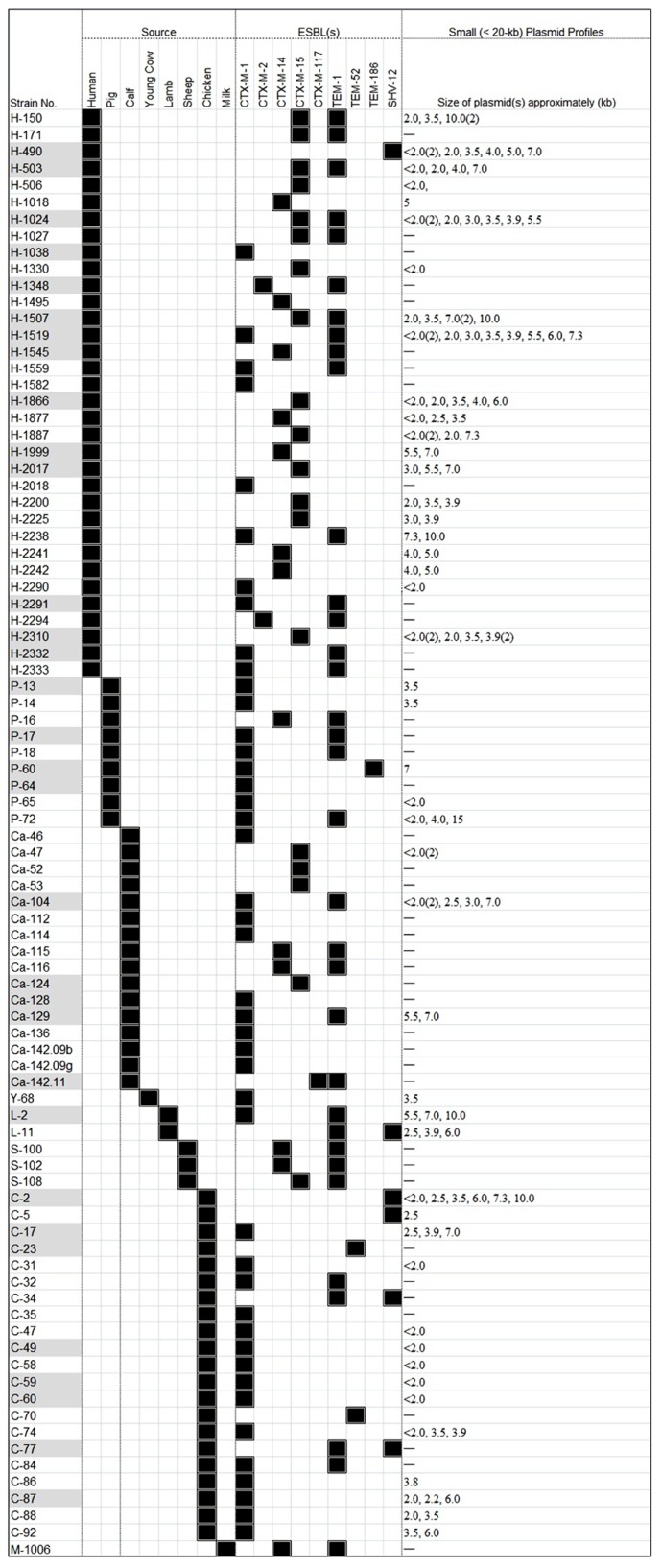
**A heat-map summary of the original 87 ESBL-positive *E. coli* isolates previously reported from healthy food-producing animals and humans in Switzerland.** Black squares shown indicate a feature present in that isolate denoting its original source and the ESBL marker(s) detected; and white squares denote features that are lacking in the corresponding bacterial isolate. The approximate sizes of small plasmids are also showing in separate columns. The symbol “–” signifies no small (<30-kb) plasmid in the corresponding strain. Isolate numbers denoted by the grayed boxes on the left of the heat-map indicate those bacteria that were selected for conjugation experiments and S1-nuclease-based PFGE studies (as described in **Figure 1**).

Plasmid DNAs from all isolates were purified using the WizardR Plus SV DNA Purification System (Promega, Madison, WI, USA) according to the manufacturer’s instructions, followed by separation in a 0.8% (w/v) agarose gel (SeaKemR LE Agarose, Lonza Wokingham Ltd, UK) staining with GelRed^TM^ (Biotium, Hayward, CA, USA). S1-nuclease (Promega, Madison, WI, USA) digestion as well as PFGE analysis were performed for selected 32 isolates based on their small plasmid subgroups and corresponding ESBL genotypes reported previously (the complete isolate information is listed in **Figure A1** in Appendix). Briefly, the procedure included a lysis step of the bacterial cells embedded in agarose plugs followed by digestion with 8 U S1 nuclease at 37°C for 45 min. Finally, each plasmid sample was resolved by PFGE in a Chef-Mapper^®^ XA System (Bio-Rad, USA) at 14°C, with a switch time between 1 and 12 s, at 6 V/cm on a 120° angle in 0.5× TBE buffer for 18 h. Each DNA band was considered a unit length of linear plasmid (Barton et al., 1995). The approximate molecular mass of plasmids was determined by comparing with *E. coli* 39R 861, containing four reference plasmids, of known molecular weights 6.9-, 36-, 63-, and 147-kb (Macrina et al., 1978).

### PCR-BASED REPLICON TYPING

Plasmids were assigned to incompatibility groups on the basis of the presence of specific replicon sequences identified by PCR using the primers previously designed and the corresponding amplification protocols described (Carattoli et al., 2005).

### CONJUGATION-BASED MATING EXPERIMENTS AND VERIFICATION

Thirty-two selected isolates were analyzed individually, for their ability to transfer cefotaxime resistance to a rifampicin-resistant, plasmid-free *E. coli* recipient (26R 793). Conjugation experiments were carried out using a broth mating protocol. Transconjugants were selected on Luria-Bertani (LB) agar plates (Difco Laboratories, Becton Dickinson, Sparks, MD, USA) containing 4 μg/ml cefotaxime (Thermo Fisher Scientific Inc., USA) and 100 μg/ml rifampicin (Sigma, Dublin, Ireland). Transfer frequencies were calculated per donor cell. Susceptibility tests were performed to confirm the plasmid transfer as described below, followed by S1-nuclease PFGE and PCR-based replicon typing (PBRT) to test which plasmids and resistance markers were transferred.

### ANTIMICROBIAL SUSCEPTIBILITY TESTING

Donor strains and the corresponding transconjugants were tested for their susceptibility to a panel of antimicrobial compounds by disk diffusion, following recommendations of the Clinical and Laboratory Standards Institute (CLSI). Susceptibility/resistance was interpreted according to the CLSI document M100-S21(CLSI, 2011). The panel of antibiotic-containing disks (Becton, Dickinson, USA) along with their abbreviated names consisted of ampicillin (AM), amoxicillin–clavulanic acid (AMC), chloramphenicol (C), ciprofloxacin (CIP), cefpodoxime (CPD), cefoxitin (FOX), cephalothin (KF), tetracycline (TE), streptomycin (S), imipenem (IPM), nalidixic acid (NA), trimethoprim–sulfamethoxazole (SXT), and trimethoprim (W). *E. coli* ATCC^TM^ 25922 was included as a quality control strain. The strains were classified as susceptible or resistant to each antimicrobial agent. Strains giving *intermediate* values were considered susceptible. In addition, minimal inhibitory concentrations (MICs) of cefotaxime for donors and transconjugants combinations were determined using E-test strips (AB Biodisk, Solna, Sweden).

### DETECTION OF RESISTANCE GENES BY MICROARRAY (GENE CHIP)

A DNA microarray analysis (AMR-ve Genotyping Kit, Clondiag Chip Technologies, Jena, Germany) was performed with the donor strains to detect the genes encoding resistance to aminoglycosides, β-lactams, chloramphenicol, erythromycin, quinolone, sulphonamides, tetracycline, trimethoprim, and an integrase-encoding gene (*intI*).

### PHYLOGENETIC CLASSIFICATION

Donor isolates that transferred cefotaxime resistance to *E. coli* 26R 793 were classified into the four main phylogenetic groups (A, B1, B2, and D) using a previously described triplex PCR-based protocol (Clermont et al., 2000). Purified DNA served as a template. Thereafter, three specific primer sets (custom-synthesized by MWG-Biotech AG, Ebersberg, Germany) were used to identify the following markers; *chuA* (279 bp), *yjaA* (211 bp), and *tspE4* (152 bp; Clermont et al., 2000).

### MULTI-LOCUS SEQUENCE TYPING

Internal amplicons of seven housekeeping genes (*adk*, *fumC*, *gyrB*, *icdF*, *mdh*, *purA*, *recA*) from the donor strains were sequenced (Wirth et al., 2006); alleles as well as sequence types (ST) were verified using the *E. coli* MLST website^1^

## RESULTS

### PLASMID PROFILING

Eighty-seven *E. coli* isolates were included in this study and low molecular weight (<20-kb) plasmids could be identified by purification and conventional agarose gel electrophoresis. About half of the isolates (*n* = 46; 53%) carried several plasmids ranging in size from approximately 2- to 20-kb, and 41 isolates (47%) were devoid of small plasmids.

**FIGURE 1:**
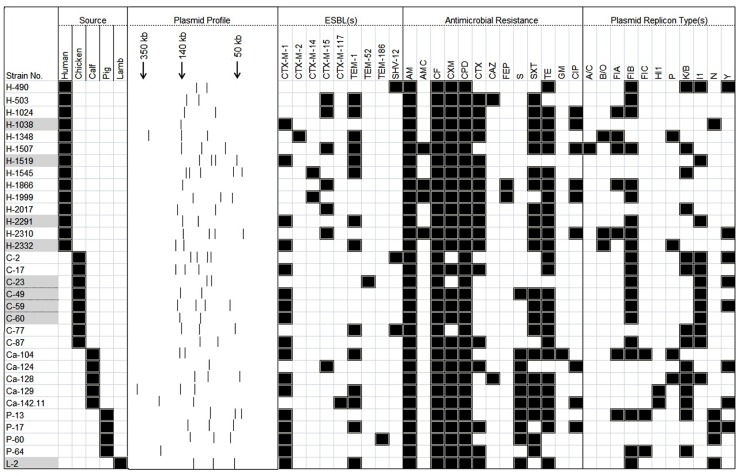
**A heat-map summary of the sources; a schematic showing the S1 nuclease plasmid profile; ESBL present/absent profiles; the resistance profile and the corresponding Inc plasmid type(s) for all 32 ESBL-positive *E. coli* previously reported from healthy food-producing animals and humans in Switzerland.** Black squares shown indicate a feature present in that isolate denoting its original source the ESBL marker(s) detected, its corresponding antimicrobial resistance profile and the Inc types detected. White squares denote features that are lacking in the corresponding bacterial isolate. Isolate numbers denotes by the grayed boxes on the left of the heat-map indicate those bacteria that contained self-transmissible ESBL-harboring plasmids (they are investigated further, as described in **Figure 2**). Antimicrobial compounds (it is known that many ESBL producers may appear susceptible or intermediate to certain oxyimino cephalosporins *in vitro*, if CLSI criteria are applied strictly, but do not respond to the respective therapies. Consequently, for clinical reporting these results have to be corrected to “resistant.”) used are abbreviated as follows: AM, ampicillin; AMC, amoxicillin–clavulanic acid; CAZ, ceftazidime; CF, cephalothin; CPD, cefpodoxime; CIP, ciprofloxacin; CTX, cefotaxime; CXM, cefuroxime; FEP, cefepime; GM, gentamicin; S, streptomycin; SXT, trimethoprim-sulfamethoxazole; TE, tetracycline.

Thirty-two isolates from the original set of 87 were selected for further study, based on their diverse plasmid profiles and the corresponding ESBL genotypes, reported previously (Geser et al., 2012a, b). All were assessed for the presence of large plasmids (ranging in size from 30- to 600-kb) by S1 nuclease digestion followed by PFGE (Barton et al., 1995). S1 nuclease plasmid analysis revealed that all 32 contained detectable large plasmids; most possessed two plasmids (*n* = 13, 40.6%) and some (*n* = 9, 28.1%) had three plasmids. Heterogeneity among the profiles was a common feature noted, although most of the plasmid profiles were related among strains isolated from particular sources. Majority of the human isolates (*n* = 12, 86%) carried two or more large (≥20-kb) plasmids and a similar situation was noted for isolates cultured from the animal sources (*n* = 16, 89%; **Figure 1**).

### PBRT OF ISOLATES EXPRESSING AN ESBL-PHENOTYPE

Eighteen plasmid replicons were detected by qualitative PBRT (Carattoli et al., 2005) among those 32 isolates carrying large (≥20-kb) plasmids. Replicon typing was identified 11 of 18 replicons. Interestingly, IncFIIA, IncFrep, IncHI2, IncL/M, IncW, IncT, or IncX types could not be detected, by PCR analysis in our collection. Nine Inc-types were identified among the isolates cultured from healthy humans. Of these, IncFIB (*n* = 9), IncFIA (*n* = 5), and IncK/B (*n* = 4) replicon types were the predominated types. Isolates recovered from poultry group had the highest number of IncI1 (*n* = 8) types. Three other replicon types were also detected in *E. coli* isolates cultured form poultry and these included IncFIB (*n* = 7), IncK/B (*n* = 4), and IncY (*n* = 3). When compared with the Inc-types identified in isolates cultured from humans, replicon types from the animal strains were less diverse. Several isolates were positive for more than one replicon type. The reason could be that they possessed multiple plasmids, or a single plasmid-encoded replication, or partitioning genes from more than one replicon family. A summary of these features along with the corresponding antimicrobial resistance profiles for all 32 isolates is shown as a heat-map in **Figure 1.**

### CONJUGATION EXPERIMENTS WITH ESBL-PRODUCING ISOLATES

Each isolate of the 32 selected *E. coli*, elaborating an ESBL-phenotype, were tested for their ability to transfer the ESBL-resistant phenotype, by conjugation under laboratory conditions. Nine isolates transferred the cefotaxime resistance marker to a susceptible *E. coli* recipient with transfer rates ranging from 3.1 × 10^-^^2^ (in the case of L-2) to 5.7 × 10^-^^1^ (for C-49) transconjugants per donor cell. Transconjugants recovered were characterized as described below.

### HORIZONTAL TRANSFER OF ANTIMICROBIAL RESISTANCE AND ASSOCIATED DETERMINANTS

**FIGURE 2:**
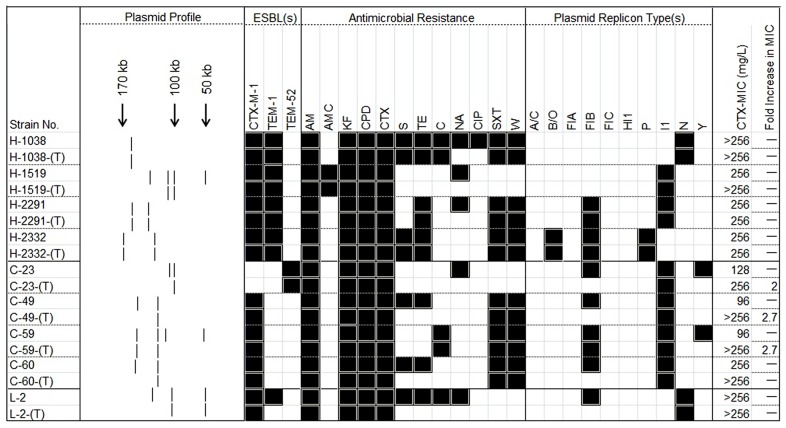
**A heat-map showing the comparison of ESBL-positive *E. coli* donors and the resultant transconjugants, characterized on the basis of their plasmid profiles; ESBL-markers identified by PCR; antimicrobial resistance profile; and plasmid replicon type(s).** The MIC for cefotaxime in each pairwise combination is also shown along with the fold increase in this value. (T) in the first column typifies the transconjugant. Black and white squares denote the presence and absence, respectively of a particular feature. The symbol “–” signifies no fold-change in the MIC for the transconjugant. Antimicrobial compounds are abbreviated as follows: AM, ampicillin; AMC, amoxicillin–clavulanic acid; C, chloramphenicol; CPD, cefpodoxime; CIP, ciprofloxacin; CTX, cefotaxime; KF, cephalothin; NA, nalidixic acid; S, streptomycin; SXT, trimethoprim-sulfamethoxazole; TE, tetracycline; W, trimethoprim.

With the exception of *E. coli* H-1519 cultured from a human and which contained two small plasmids (of 2- and 7-kb; data not shown), none of the other smaller (<30-kb) plasmids could be transferred to the *E. coli* rifampicin-resistant recipient under laboratory conditions. Four isolates (H-2291, H-2332, C-59, and L-2) were identified wherein two large plasmids were transferred *via* conjugation and a further four (H-1038, C-23, C-49, and C-60) transferred a single large plasmid only (**Figure 2**).

Conjugation studies and plasmid profile analysis of all transconjugants showed that the nine isolates possessed 16 plasmids. Of these, 14 were large (>30-kb) plasmids and the replicon types were also identified, by PCR. Six plasmids belonged to different donors and possessed IncI1 replicons. Three large plasmids contained IncFIB replicons and two contained IncN types. Single plasmids were typed as being members of the IncP or IncB/O replicon families (**Figure 2**).

Following conjugation the antimicrobial susceptibility of all nine transconjugants was determined by disk diffusion. In addition to cefotaxime resistance, resistance to several non-β-lactam-based antimicrobial compounds such as chloramphenicol, tetracycline, trimethoprim, and trimethoprim–sulfamethoxazole were also transferred to the recipient, suggesting that these markers are genetically linked. The MIC values recorded for all nine donors ranged from 96 to >256 μg/ml by cefotaxime E-test. When these MIC values were measured for the transconjugants, all were recorded at 256 g/ml and in three cases there was between a 2- to 2.7-fold increase, in this value the MICs of transconjugants correspondingly were higher (>256 μg/ml; **Figure 2**).

Twenty-eight antibiotic resistance-encoding genes were tested on commercial microarray in all nine donor strains. Only nine of these markers, including *aadA*, *cat*, *ctx*, *dfr*, *tem*, *tet*, *strA*, *strB*, and *sul* were detected. A CTX-encoding gene was detected in eight isolates, with an additional isolate positive for the TEM gene. These data were subsequently confirmed by PCR and the genes identified as *bla*_CTX-M-1_ and *bla*_TEM-52_, respectively. Five strains were positive for the *bla*_TEM-1_ gene. Six isolates were positive for the integrase-encoding gene (*intl*) associated with class 1 integrons. The genotypes of all nine donors and their corresponding transconjugants are summarized in **Figure 2** and **Table 1**.

**Table 1:** Summary of genotypic and phenotypic characteristics of nine ESBL–producing *E. coli* isolates cultured from healthy food–producing animals and humans in Switzerland.

Isolate no.	Phylogenetic groups	Serotype	MLST	Plasmid(s) (kb) isolated from transconjugants	Additional resistance determinants identified using a commercial microarray^a^
H–1038	B1	O8:H21	Unknown ST	140-	*aadA-strA-strB-cat-dfr-sul-tet-intl*
H-1519	A	O2:H48	ST10 complex	2-, 7-, 80-, 100-	*–*
H-2291	A	O53:H18	ST1638	110-, 140-	*aadA-dfr-sul-tet-intl*
H–2332	D	O24:H26	ST57/ST350 complex	100-, 150-	*aadA-strA-strB-cat-dfr-sul-tet-intl*
C–23	B1	O3:Hnt	ST1389	100-	–
C-49	B1	Ont:H21	ST446 complex	110-	*aadA-strA-strB-dfr-sul-tet-intl*
C-59	B1	O86:H51	ST155 complex 1	110-, 150-	*aadA-cat-dfr-sul-tet-intl*
C-60	B1	O68:H21	ST3174 complex	110-	*aadA-strA-strB-dfr-sul-tet-intl*
L-2	D	Ont:H11	ST295	50-, 100-	*strB-cat-sul-tet*

a Method used: Identi Bac Chip (AMR-ve Genotyping Kit, Version 05m); the symbol “–” signifies the fact that the bacterial isolate is negative for those genes included on the commercial microarray; “Ont” denotes an isolate whose serotype was non-typable.

### GENOTYPING AND PHYLOGENETIC CLASSIFICATION OF NINE DONORS

All donor isolates were sub-typed by MLST and eight STs and an unknown ST were identified (**Table 1**). Genotypings identified were ST1389, ST604 (ST446 complex), ST155 (ST155 complex), ST3174, ST295, ST10 (ST10 complex), ST1638, ST57 (ST350 complex). The allele numbers of the unknown ST type were as follows: *adk* 6, *fumC* 4, *gyrB* 3, *icdF* 1, *mdh* 20, *purA* 8, *recA* 44.

The nine strains harboring self-transmissible plasmids could be assigned to phylogenetic groups A, B1, and D. Five isolates were assigned to group B1 and two assigned to group A. While two isolates were assigned to the D phylogenetic groups and non-isolate belonged to group B2 (**Table 1**).

## DISCUSSION

By definition, plasmids do not carry genes essential for the growth of host cells under non-stressed conditions but most plasmids confer positively selectable phenotypes including antimicrobial resistance genes among others (Carattoli, 2011). Plasmids represent an important pool of adaptive and transferable genetic information and large plasmids (defined as being >30-kb in size) are commonly being capable of transferring their genetic information in bacteria (Norman et al., 2009; Smillie et al., 2010; Williams et al., 2013). Previously *E. coli* isolates were cultured from a number of healthy food-producing animals and humans in Switzerland and all expressed an ESBL-phenotype. In the collection of 32 isolates which were further studied, 82 megaplasmids were identified. Four plasmids had molecular sizes ranging from 200- to about 350-kb and a further 78 (95%) were large plasmids of sizes between 50- and 200-kb were observed. The approximate molecular sizes of plasmids were determined by comparing with the reference plasmids (Macrina et al., 1978). At the outset, these data suggested that the ESBL markers may be mapped to larger sized plasmids (50- to 200-kb).

Currently, 27 Inc groups are recognized among the Enterobacteriaceae family (Carattoli, 2011). Particular plasmid incompatibility groups are more frequently encountered among *E. coli* and these play a major role in the dissemination of specific resistance genes. For instance, in Netherlands, genetically related IncI1 plasmids carrying the *bla*_CTX-M-1_ gene were identified in *E. coli* isolates from poultry meat samples and in infected individuals suggesting that transfer of these plasmids may have occurred among strains from food-producing animals and humans (Leverstein-van Hall et al., 2011). CTX-M-1 was also identified on plasmids belonging to the IncN family in Europe among porcine (Moodley and Guardabassi, 2009), canine (Schink et al., 2011), and human *E. coli* isolates (Novais et al., 2007). In contrast, IncFII, IncA/C, IncL/M, IncN, and IncI1 plasmids carrying ESBL determinants are currently considered to be *epidemic resistance plasmids*, being detected in Enterobacteriaceae of different origin and sources (Carattoli, 2011). In the study reported here, PBRT typing identified 11 of 18 replicons (including IncA/C, IncB/O, IncFIA, IncFIB, IncFIC, IncHI1, IncP, IncK/B, IncI1, IncN, and IncY). IncFIB, IncK/B, and IncI1 were the more commonly identified replicon families in this study with 67% (*n* = 8) being IncI1 plasmids and which were isolated from the poultry samples. Moreover, conjugative transfer of plasmids belonging to incompatibility groups IncI1, IncFIB, and, less frequently, IncN, IncB/O, and IncP could be demonstrated under laboratory conditions, suggesting that the potential dissemination of these markers may exist in the wildlife (**Figure 2**). This observation is interesting given that plasmids of IncI (both IncI1 and IncI2) and IncF groups are commonly recovered from *E. coli* and *Salmonella* species cultured from both animals and humans, and they are also linked to a number of genes coding for ESBL-genotypes (Johnson et al., 2007; Carattoli, 2009; Doumith et al., 2012).

IncI1 plasmids are often associated with resistance to multiple antimicrobial compounds, particularly extended-spectrum cephalosporinases of both the CTX-M and CMY types (Hopkins et al., 2006; Marcade et al., 2009). These have also been linked to TEM-52 enzymes. IncI1 plasmids producing TEM-52 have been identified in *E. coli* cultured from chicken and turkey meat in Denmark and the UK (Jensen et al., 2006; Randall et al., 2011). Interestingly an IncI1 plasmid carrying *bla*_CTX-M-15_ and *bla*_TEM-1_ was associated with the recent 2011 outbreak of the *E. coli* O104:H4 in Germany^2^. Three conjugative IncI1 plasmids were identified among the *E. coli* isolates in this study. One such plasmid was from a healthy chicken isolate and contained a *bla*_TEM-52_ marker. Others may lead to the transfer of *bla*_CTX-M-1_ (**Figure 2**). These findings further support the suggestion that there may be prevalent plasmid types that are responsible for the dissemination of specific resistance genes in *E. coli* causing both community- and hospital-acquired infections (Sherley et al., 2003; Hopkins et al., 2006; Novais et al., 2007).

IncF replicons (including FIA, FIB, and FIC) were the most frequently detected replicon types in the studied collection (45%). Interestingly, no IncFII type was identified. These data are in accordance with the previous reports (Johnson et al., 2007; Marcade et al., 2009). IncF replicons are widely distributed among *E. coli* and these plasmid types seem to be well adapted to this species. Three large potential conjugative plasmids (100- to 150-kb) of IncFIB replicon type were identified. This finding supports the view that IncF family plasmids play a major role in the dissemination of antibiotic resistance in Enterobacteriaceae, and some have been associated with specific genes conferring resistance to aminoglycosides, β-lactams, and quinolones (Hopkins et al., 2006; Marcade et al., 2009). Plasmids of the IncP group are recognized as a significant antibiotic resistance marker reservoir due to their self-transmissibility and broad host range (Schluter et al., 2007). Only a single possible conjugative IncP plasmid was identified among the transmissible plasmids studied. A similar situation was noted for the IncB/O type. This feature was interesting, given the fact that in previous studies IncP and IncB/O were the dominant Inc-types identified among transmissible plasmids characterized (Karczmarczyk et al., 2011).

β-Lactamase-encoding (*bla*) genes have benefited from the various mechanisms available to promote HGT between bacteria, thereby ensuring the spread of these markers to new hosts. It is often included as component parts of multi-resistance plasmids commonly found in clinical isolates (Bush and Jacoby, 2010). Identification of the resistance genes together with an investigation of their potential for dissemination among bacteria of animal origin provides valuable insights into this important resistance gene pool (Norman et al., 2009). Use of extended-spectrum cephalosporins, in animal and human medicine can select for ESBL-genotypes. Dating back to the late 1990s, CTX-M enzymes are regarded as the predominant ESBL-types of animal origin as well as in human isolates in Europe (Livermore et al., 2007). As TEM-1 is the most common plasmid-mediated β-lactamase identified in enteric Gram-negative bacilli, high rates of ampicillin-resistant, *bla*_TEM_-positive isolates are to be expected (Paterson and Bonomo, 2005). In the current study, the *bla*_CTX-M-1_ gene was identified in eight transconjugants (including four from healthy humans, three from chickens, and one from a lamb; **Figure 2**). The *bla*_TEM-1_ gene was also identified in five transconjugants (four from healthy humans and one from a chicken sample; **Figure 2**). To our knowledge, neither *bla*_CTX-M-1_ nor *bla*_TEM-1_ associated with conjugative plasmids has been reported in healthy humans or food-producing animals in Switzerland.

In the laboratory, cefotaxime is used for optimum detection of *bla*_ESBL_-encoding isolates aiding in the identification of CTX-M carrying bacteria (Rodriguez et al., 2006). In this study, cefotaxime resistance was observed to occur concomitantly with resistance to AMP/KF/CPD in eight transconjugants and this feature could be linked directly to the presence of *bla*_CTX-M-1_ (in eight of nine transconjugants), to *bla*_TEM-1_ (in five isolates), and to *bla*_TEM-52_ (in one isolate). However, only one –isolate showed resistance to amoxicillin–clavulanic acid (H-1519). As no evidence for mechanistic resistance, such as the expression of inhibitor-resistant TEM or SHV enzymes of the Bush–Jacoby groups 2be or 2ber (Bush and Jacoby, 2010) was detected (data not shown), this phenotype was most likely due to the hyper-expression of co-linear TEM-1 markers (Ortega et al., 2012; Zurfluh et al., 2013). Interestingly, the MICs of cefotaxime of three transconjugants [C-23-(T); C-49-(T) and C-59-(T), **Figure 2**] were between 2- and 2.7-fold higher than the corresponding donor strains. These increases indicated that an amplification of multiple copies of the ESBL gene or occurrence of a point mutation in one gene copy might increase specific activity of β-lactamase or downregulate its cell wall porins to increase resistance (Tenover, 2006; Sun et al., 2009).

All nine transconjugants showed phenotypic resistance to more than one class of antimicrobial compound. One isolate [H-1038-(T); **Figure 2**] was found to be resistant to members of four different drug classes (aminoglycosides, chloramphenicol, sulfamethoxazole/trimethoprim, and tetracycline), and a further three [H-2291-(T), H-2332-(T) and C-59-(T), **Figure 2**] were resistant to three different antimicrobial compound classes (aminoglycosides, sulfamethoxazole/trimethoprim, and tetracycline or chloramphenicol). To our knowledge, this is the first evidence of this emerging multi-resistant clone with large conjugative plasmids from healthy humans and animals in Switzerland.

Recently, ESBL-producing *E. coli* ST131 and ST1722 were cultured from rivers and lakes (Zurfluh et al., 2013) and ST61 was recovered from slaughterhouses (Endimiani et al., 2012) in Switzerland. Furthermore, ESBL-producing *E. coli* ST57 and ST371 were cultured from chickens in the UK, Germany, and Canada (Randall et al., 2011). Of the nine donor strains investigated in this study, the clonal lineages detected among healthy humans included phylogroup B1-O8:H21-unknown ST type, phylogroup A-O53:H18-ST1638, a pathogenic phylogroup D-O24:H26-ST57/ST350 complex, and phylogroup A-O2:H48-ST10 complex. Among the five animal isolates the lineages included phylogroup B1-O3:Hnt-ST1389, phylogroup B1-Ont:H21-ST446 complex, phylogroup B1-O68:H21-ST3174 complex, phylogroup B1-O86:H51-ST155 complex, and phylogroup D-Ont:H11-ST295 from a lamb source (**Table 1**). Our study represents the first report of ESBL-producing *E. coli* of nine different ST types from animals and humans in Switzerland. Interestingly, these showed a distribution of plasmid replicon types similar to those reported previously in unselected *E. coli*, whereas in contrast the distribution of STs was markedly different (EFSA Panel on Biological Hazards [BIOHAZ], 2011; Randall et al., 2011). These finding may indicate the presence of systems affecting the incorporation and/or maintenance of antibiotic resistance genes not related to plasmid restriction (Bengtsson et al., 2012).

Characterizing plasmids from bacteria of different origins may provide early insights into the epidemiology of important resistance markers, including ESBL-determinants. Few studies have provided clear evidence of a direct transmission of ESBL-producing *E. coli* isolates from food-producing animals and/or food to humans. Nonetheless, these data showed that MDR phenotypes, including resistance to newer generations of antimicrobial compounds, were transferable and in some cases were directly linked to large conjugative plasmids carrying multiple resistance genes. This situation could, in the future give rise to the co-selection, promoting the dissemination of ESBL-genotypes among bacteria of healthy food-producing animal and human origins alike.

## Conflict of Interest Statement

The authors declare that the research was conducted in the absence of any commercial or financial relationships that could be construed as a potential conflict of interest.

## Footnotes

1 http://mlst.ucc.ie/mlst/dbs/Ecoli

2 www.efsa.europa.eu/en/efsajournal/doc/2274.pdf

## References

[B1] Barton B. M. Harding G. P. Zuccarelli A. J. (1995) A general method for detecting and sizing large plasmids. *Anal. Biochem.* 226 235–240 10.1006/abio.1995.1220 7793624

[B2] Bengtsson S. Naseer U. Sundsfjord A. Kahlmeter G. Sundqvist M. (2012) Sequence types and plasmid carriage of uropathogenic *Escherichia coli* devoid of phenotypically detectable resistance. *J. Antimicrob. Chemother.* 67 69–73 10.1093/jac/dkr421 21980069

[B3] Boyd E. F. Hill C. W. Rich S. M. Hartl D. L. (1996) Mosaic structure of plasmids from natural populations of *Escherichia coli*. *Genetics* 143 1091–1100 8807284 10.1093/genetics/143.3.1091 PMC1207381

[B4] Bush K. (2010) Bench-to-bedside review: the role of beta-lactamases in antibiotic-resistant Gram-negative infections. *Crit. Care* 14 224 10.1186/cc8892 PMC2911681 20594363

[B5] Bush K. Jacoby G. A. (2010) Updated functional classification of beta-lactamases. *Antimicrob. Agents Chemother.* 54 969–976 10.1128/AAC.01009-09 19995920 PMC2825993

[B6] Carattoli A. (2009) Resistance plasmid families in Enterobacteriaceae. *Antimicrob. Agents Chemother.* 53 2227–2238 10.1128/AAC.01707-08 19307361 PMC2687249

[B7] Carattoli A. (2011) Plasmids in Gram negatives: molecular typing of resistance plasmids. *Int. J. Med. Microbiol.* 301 654–658 10.1016/j.ijmm.2011.09.003 21992746

[B8] Carattoli A. Bertini A. Villa L. Falbo V. Hopkins K. L. Threlfall E. J. (2005) Identification of plasmids by PCR-based replicon typing. *J. Microbiol. Methods* 63 219–228 10.1016/j.mimet.2005.03.018 15935499

[B9] Clermont O. Bonacorsi S. Bingen E. (2000) Rapid and simple determination of the *Escherichia coli* phylogenetic group. *Appl. Environ. Microbiol.* 66 4555–4558 10.1128/AEM.66.10.4555-4558.2000 11010916 PMC92342

[B10] CLSI. (2011) *Performance Standards for Antimicrobial Susceptibility Testing: 21st Informational Supplement*. CLSI Document M100-S21. Wayne, PA: Clinical and Laboratory Standards Institute

[B11] Coque T. M. Baquero F. Canton R. (2008) Increasing prevalence of ESBL-producing Enterobacteriaceae in Europe. *Euro. Surveill.* 13 19021958

[B12] Doumith M. Dhanji H. Ellington M. J. Hawkey P. Woodford N. (2012) Characterization of plasmids encoding extended-spectrum beta-lactamases and their addiction systems circulating among *Escherichia coli* clinical isolates in the UK. *J. Antimicrob. Chemother.* 67 878–885 10.1093/jac/dkr553 22210753

[B13] EFSA Panel on Biological Hazards (BIOHAZ)(2011) (Scientific opinion on the public health risks of bacterial strains producing extended-spectrum β-lactamases and/or AmpC β-lactamases in food and food-producing animals. *EFSA J.* 9 2322 10.2903/j.efsa.2011.2322

[B14] Endimiani A. Rossano A. Kunz D. Overesch G. Perreten V. (2012) First countrywide survey of third-generation cephalosporin-resistant *Escherichia coli* from broilers, swine, and cattle in Switzerland. *Diagn. Microbiol. Infect. Dis.* 73 31–38 10.1016/j.diagmicrobio.2012.01.004 22578936

[B15] Foxman B. (2013) Editorial commentary: extended-spectrum beta-lactamase-producing *Escherichia coli* in the United States: time to rethink empirical treatment for suspected *E.coli infections?* *Clin. Infect. Dis.* 56 649–651 10.1093/cid/cis947 23150212

[B16] Garcillan-Barcia M. P. Alvarado A. De La Cruz F. (2011) Identification of bacterial plasmids based on mobility and plasmid population biology. *FEMS Microbiol. Rev.* 35 936–956 10.1111/j.1574-6976.2011.00291.x 21711366

[B17] Geser N. Stephan R. Hachler H. (2012a) Occurrence and characteristics of extended-spectrum beta-lactamase (ESBL) producing Enterobacteriaceae in food producing animals, minced meat and raw milk. *BMC Vet. Res.* 8 21 10.1186/1746-6148-8-21 PMC3319423 22397509

[B18] Geser N. Stephan R. Korczak B. M. Beutin L. Hachler H. (2012b) Molecular identification of extended-spectrum-beta-lactamase genes from Enterobacteriaceae isolated from healthy human carriers in Switzerland. *Antimicrob. Agents Chemother.* 56 1609–1612 10.1128/AAC.05539-11 22155836 PMC3294945

[B19] Harajly M. Khairallah M. T. Corkill J. E. Araj G. F. Matar G. M. (2010) Frequency of conjugative transfer of plasmid-encoded ISEcp1 – blaCTX-M-15 and aac(6^′^)-lb-cr genes in Enterobacteriaceae at a tertiary care center in Lebanon – role of transferases. *Ann. Clin. Microbiol. Antimicrob.* 9 19 10.1186/1476-0711-9-19 PMC2919444 20646305

[B20] Hopkins K. L. Liebana E. Villa L. Batchelor M. Threlfall E. J. Carattoli A. (2006) Replicon typing of plasmids carrying CTX-M or CMY beta-lactamases circulating among *Salmonella* and *Escherichia coli* isolates. *Antimicrob. Agents Chemother.* 50 3203–3206 10.1128/AAC.00149-06 16940132 PMC1563510

[B21] Jensen L. B. Hasman H. Agerso Y. Emborg H. D. Aarestrup F. M. (2006) First description of an oxyimino-cephalosporin-resistant, ESBL-carrying *Escherichia coli* isolated from meat sold in Denmark. *J. Antimicrob. Chemother.* 57 793–794 10.1093/jac/dkl048 16504999

[B22] Johnson T. J. Wannemuehler Y. M. Johnson S. J. Logue C. M. White D. G. Doetkott C. et al. (2007) Plasmid replicon typing of commensal and pathogenic *Escherichia coli* isolates. *Appl. Environ. Microbiol.* 73 1976–1983 10.1128/AEM.02171-06 17277222 PMC1828809

[B23] Karczmarczyk M. Walsh C. Slowey R. Leonard N. Fanning S. (2011) Molecular characterization of multidrug-resistant *Escherichia coli* isolates from Irish cattle farms. *Appl. Environ. Microbiol.* 77 7121–7127 10.1128/AEM.00601-11 21856840 PMC3194884

[B24] Leverstein-van Hall M. A. Dierikx C. M. Cohen Stuart J. Voets G. M. Van Den Munckhof M. P. Van Essen-Zandbergen A. et al. (2011) Dutch patients, retail chicken meat and poultry share the same ESBL genes, plasmids and strains. *Clin. Microbiol. Infect.* 17 873–880 10.1111/j.1469-0691.2011.03497.x 21463397

[B25] Livermore D. M. Canton R. Gniadkowski M. Nordmann P. Rossolini G. M. Arlet G. (2007) CTX-M: changing the face of ESBLs in Europe. *J. Antimicrob. Chemother.* 59 165–174 10.1093/jac/dkl483 17158117

[B26] Macrina F. L. Kopecko D. J. Jones K. R. Ayers D. J. Mccowen S. M. (1978) A multiple plasmid-containing *Escherichia coli* strain: convenient source of size reference plasmid molecules. *Plasmid* 1 417–420 10.1016/0147-619X(78)90056-2 372973

[B27] Marcade G. Deschamps C. Boyd A. Gautier V. Picard B. Branger C. (2009) Replicon typing of plasmids in *Escherichia coli* producing extended-spectrum beta-lactamases. *J. Antimicrob. Chemother.* 63 67–71 10.1093/jac/dkn428 18931389

[B28] Moodley A. Guardabassi L. (2009) Transmission of IncN plasmids carrying bla(CTX-M-1) between commensal *Escherichia coli* in pigs and farm workers. *Antimicrob. Agents Chemother.* 53 1709–1711 10.1128/AAC.01014-08 19188380 PMC2663060

[B29] Norman A. Hansen L. H. Sorensen S. J. (2009) Conjugative plasmids: vessels of the communal gene pool. *Philos. Trans. R. Soc. Lond. B Biol. Sci.* 364 2275–2289 10.1098/rstb.2009.0037 19571247 PMC2873005

[B30] Novais A. Canton R. Moreira R. Peixe L. Baquero F. Coque T. M. (2007) Emergence and dissemination of Enterobacteriaceae isolates producing CTX-M-1-like enzymes in Spain are associated with IncFII (CTX-M-15) and broad-host-range (CTX-M-1, -3, and -32) plasmids. *Antimicrob. Agents Chemother.* 51 796–799 10.1128/AAC.01070-06 17145793 PMC1797763

[B31] Ortega A. Oteo J. Aranzamendi-Zaldumbide M. Bartolome R. M. Bou G. Cercenado E. (2012) Spanish multicenter study of the epidemiology and mechanisms of amoxicillin-clavulanate resistance in *Escherichia coli*. *Antimicrob. Agents Chemother.* 56 3576–3581 10.1128/AAC.06393-11 22491692 PMC3393459

[B32] Paterson D. L. Bonomo R. A. (2005) Extended-spectrum beta-lactamases: a clinical update. *Clin. Microbiol. Rev.* 18 657–686 10.1128/CMR.18.4.657-686.2005 16223952 PMC1265908

[B33] Randall L. P. Clouting C. Horton R. A. Coldham N. G. Wu G. Clifton-Hadley F. A. (2011) Prevalence of *Escherichia coli* carrying extended-spectrum beta-lactamases (CTX-M and TEM-52) from broiler chickens and turkeys in Great Britain between 2006 and 2009. *J. Antimicrob. Chemother.* 66 86–95 10.1093/jac/dkq396 21098542

[B34] Rodriguez I. Rodicio M. R. Mendoza M. C. Cruz Martin M. (2006) Large conjugative plasmids from clinical strains of *Salmonella enterica* serovar Virchow contain a class 2 integron in addition to class 1 integrons and several non-integron-associated drug resistance determinants. *Antimicrob. Agents Chemother.* 50 1603–1607 10.1128/AAC.50.4.1603-1607.2006 16569896 PMC1426967

[B35] Rogers B. A. Sidjabat H. E. Paterson D. L. (2011) *Escherichia coli* O25b-ST131: a pandemic, multiresistant, community-associated strain. *J. Antimicrob. Chemother.* 66 1–14 10.1093/jac/dkq415 21081548

[B36] Schink A. K. Kadlec K. Schwarz S. (2011) Analysis of bla(CTX-M)-carrying plasmids from *Escherichia coli* isolates collected in the BfT-GermVet study. *Appl. Environ. Microbiol.* 77 7142–7146 10.1128/AEM.00559-11 21685166 PMC3194854

[B37] Schluter A. Szczepanowski R. Puhler A. Top E. M. (2007) Genomics of IncP-1 antibiotic resistance plasmids isolated from wastewater treatment plants provides evidence for a widely accessible drug resistance gene pool. *FEMS Microbiol. Rev.* 31 449–477 10.1111/j.1574-6976.2007.00074.x 17553065

[B38] Sherley M. Gordon D. M. Collignon P. J. (2003) Species differences in plasmid carriage in the Enterobacteriaceae. *Plasmid* 49 79–85 10.1016/S0147-619X(02)00107-5 12584004

[B39] Smillie C. Garcillan-Barcia M. P. Francia M. V. Rocha E. P. De La Cruz F. (2010) Mobility of plasmids. *Microbiol. Mol. Biol. Rev.* 74 434–452 10.1128/MMBR.00020-10 20805406 PMC2937521

[B40] Stalder T. Barraud O. Casellas M. Dagot C. Ploy M. C. (2012) Integron involvement in environmental spread of antibiotic resistance. *Front. Microbiol* 3: 119 10.3389/fmicb.2012.00119 PMC3321497 22509175

[B41] Sun S. Berg O. G. Roth J. R. Andersson D. I. (2009) Contribution of gene amplification to evolution of increased antibiotic resistance in *Salmonella typhimurium*. *Genetics* 182 1183–1195 10.1534/genetics.109.103028 19474201 PMC2728858

[B42] Tenover F. C. (2006) Mechanisms of antimicrobial resistance in bacteria. *Am. J. Med.* 119 S3–S10 10.1016/j.amjmed.2006.03.011 16735149

[B43] van Hoek A. H. Mevius D. Guerra B. Mullany P. Roberts A. P. Aarts H. J. (2011) Acquired antibiotic resistance genes: an overview. *Front Microbiol* 2: 203 10.3389/fmicb.2011.00203 PMC3202223 22046172

[B44] Williams L. E. Wireman J. Hilliard V. C. Summers A. O. (2013) Large plasmids of *Escherichia coli* and *Salmonella* encode highly diverse arrays of accessory genes on common replicon families. *Plasmid* 69 36–48 10.1016/j.plasmid.2012.08.002 22939841

[B45] Wirth T. Falush D. Lan R. Colles F. Mensa P. Wieler L. H. (2006) Sex and virulence in *Escherichia coli*: an evolutionary perspective. *Mol. Microbiol.* 60 1136–1151 10.1111/j.1365-2958.2006.05172.x 16689791 PMC1557465

[B46] Zhou Y. Y. Call D. R. Broschat S. L. (2012) Genetic relationships among 527 Gram-negative bacterial plasmids. *Plasmid* 68 133–141 10.1016/j.plasmid.2012.05.002 22587825

[B47] Zurfluh K. Hächler H. Nuesch-Inderbinen M. Stephan R. (2013) Characteristics of extended-spectrum beta-lactamase (ESBL)- and carbapenemase-producing Enterobacteriaceae isolated from rivers and lakes in Switzerland. *Appl. Environ. Microbiol.* 79 3021–3026 10.1128/AEM.00054-13 23455339 PMC3623138

